# Increase of Calcium Sensing Receptor Expression Is Related to Compensatory Insulin Secretion during Aging in Mice

**DOI:** 10.1371/journal.pone.0159689

**Published:** 2016-07-21

**Authors:** Yoon Sin Oh, Eun-Hui Seo, Young-Sun Lee, Sung Chun Cho, Hye Seung Jung, Sang Chul Park, Hee-Sook Jun

**Affiliations:** 1 Lee Gil Ya Cancer and Diabetes Institute, Gachon University, Incheon, Korea; 2 Gachon Medical Research Institute, Gil Hospital, Incheon, Korea; 3 Department of Internal Medicine, Seoul National University College of Medicine, Seoul, Korea; 4 College of Pharmacy and Gachon Institute of Pharmaceutical Science, Gachon University, Incheon, Korea; Institut d'Investigacions Biomèdiques August Pi i Sunyer, SPAIN

## Abstract

Type 2 diabetes is caused by both insulin resistance and relative insulin deficiency. To investigate age-related changes in glucose metabolism and development of type 2 diabetes, we compared glucose homeostasis in different groups of C57BL/6J mice ranging in age from 4 months to 20 months (4, 8, 12, 16 and 20 months). Interestingly, we observed that non-fasting glucose levels were not significantly changed, but glucose tolerance gradually increased by 20 months of age, whereas insulin sensitivity declined with age. We found that the size of islets and glucose-stimulated insulin secretion increased with aging. However, mRNA expression of pancreatic and duodenal homeobox 1 and granuphilin was decreased in islets of older mice compared with that of 4-month-old mice. Serum calcium (Ca^2+^) levels were significantly decreased at 12, 20 and 28 months of age compared with 4 months and calcium sensing receptor (CaSR) mRNA expression in the islets significantly increased with age. An extracellular calcium depletion agent upregulated CaSR mRNA expression and consequently enhanced insulin secretion in INS-1 cells and mouse islets. In conclusion, we suggest that decreased Ca^2+^ levels and increased CaSR expression might be involved in increased insulin secretion to compensate for insulin resistance in aged mice.

## Introduction

With the global population aging, there has been an increase in the prevalence of impaired glucose tolerance and type 2 diabetes [1–3]. A compensatory increase of beta cell function and beta cell mass to adapt to metabolic stress associated with aging and insulin resistance can maintain normal glucose levels. However, when beta cells fail and can no longer compensate, type 2 diabetes develops [4].

Insulin secreted by the pancreatic beta cells is the key hormone in regulating glucose metabolism. There exists a close relationship between insulin secretion and insulin sensitivity in the maintenance of normal glucose tolerance, such that insulin secretion as well as beta cell mass increases as insulin sensitivity decreases [5]. Therefore, an imbalance between the need for insulin and the ability to secrete insulin can cause type 2 diabetes [6]. It is believed that increased insulin resistance during the aging process results in a compensatory increase in insulin secretion, but eventually defective and decreased insulin secretion can occur [7–9]. However, the precise mechanism of age-related changes remains unknown.

Insulin secretion is a highly dynamic process regulated by complex mechanisms [10]. Systemic calcium (Ca^2+^) signaling, including intracellular Ca^2+^ levels, Ca^2+^ dependent enzymes, and Ca^2+^ channels, is one of the primary factors that regulates insulin secretion in beta cells [11]. It has been reported that the calcium sensing receptor (CaSR), which regulates calcium homeostasis as an extracellular receptor, is expressed in the beta cells of rodent and human pancreatic islets [12], and its activation markedly increases insulin secretory responses [13, 14].

In this study, male C57BL/6J mice of different ages were studied for age-related changes in glucose homeostasis and beta cell function. We found that serum Ca^2+^ levels were significantly decreased and accordingly CaSR expression was increased, which resulted in increased insulin secretion. These phenomena might contribute to the compensatory insulin secretion seen during increased insulin resistance in aged mice.

## Material and Methods

### Mice

C57BL/6J mice were obtained from the Korea Research Institute of Bioscience and Biotechnology (Daejeon, Korea). Food and water were provided *ad libitum*, and the mice were kept on a 12 h light, 12 h dark cycle. All protocols involving animal use and sacrifice were reviewed and approved by the Animal Care Committee of Gachon University. Male mice were used for all studies.

### Maintenance of INS-1 cells

INS-1 cells (passage 20 ~ 30), derived from a rat insulinoma, were maintained at 37°C (95% air/5% CO_2_) in RPMI1640 supplemented with 10% fetal bovine serum, 100 units/ml penicillin, and 100 μg/ml streptomycin.

### Monitoring blood glucose levels, body weight, and food intake

Animals were fed *ad libitum* with a standard rodent diet, and were monitored every week for the development of hyperglycemia using a glucometer. Body weight and food intake were monitored at the same time each week.

### Glucose and insulin tolerance tests

Glucose- and insulin tolerance tests were performed in different groups of mice at different ages. For glucose tolerance tests, mice were subjected to overnight fasting and were then injected with glucose (2 g/kg body weight, I.P.). A glucometer was used to measure glucose in the tail vein blood at 0, 15, 30, 60, 90, and 120 min after injection. For insulin tolerance tests, mice (fasted for 4 h) were injected with insulin (1 U/kg body weight, I.P.), followed by measurement of blood glucose levels. Changes in blood glucose were calculated as the percentage of the initial blood glucose level.

### Immunohistochemical analyses of the alpha, beta and delta cells in pancreatic islets

The pancreas from mice at different ages were fixed in 10% buffered formalin and embedded in paraffin. The tissue sections were blocked with blocking solution and then incubated with rabbit anti-insulin (1:100, Santa Cruz Biotechnology, Santa Cruz, CA), rabbit-anti-glucagon (1:100, Dako, Carpenteria, CA) and rabbit anti-somatostatin (1:100, Dako). Horseradish peroxidase-conjugated goat anti-rabbit IgG (1:500) (Chemicon) was used as the secondary antibody. Hematoxylin (Sigma, St. Louis, MO) was used as a nuclear counterstain for light microscopy, and peroxidase staining was performed with DAB as the chromogen (Dako). Quantitative evaluation of the beta cell area was performed on insulin-stained sections using the UTHSCSA Image Tool program (http://compdent.uthscsa.edu). The insulin-positive area of all islets was measured. Beta cell area was calculated by dividing the area of all insulin-positive cells by the number of islets. The numbers of islets measured were: 180 islets (4 months), 187 islets (8 months), 184 islets (12 months), and 133 islets (20 months).

### Isolation of islets from mice

Islets were isolated from C57BL/6J mice using a liberase digestion method described previously [15]. Briefly, after injection of liberase into the bile duct, the swollen pancreas was excised and incubated at 37°C. The islets were then separated by Ficoll gradient centrifugation at 2000 *g* for 10 min. Islets were collected and washed with HEPES balanced salt solution (HBSS) (114 mmol/l NaCl, 4.7 mmol/l KCl, 1.2 mmol/l KH_2_PO_4_, 1.16 mmol/l MgSO_4_, 20 mmol/l HEPES, 2.5 mmol/l CaCl_2_, 25 mmol/l NaHCO_3_, and 0.2% bovine serum albumin). Size-matched healthy islets were handpicked under a stereomicroscope.

### Glucose-stimulated insulin secretion

To examine in vivo insulin secretion, blood samples were collected at 0 and 30 min after glucose injection (2 g/kg body weight). After centrifugation at 3000 g for 20 min, serum was collected. INS-1 cells or isolated islets (8 islets/group) from each age group were plated on 24-well plates and incubated with HBSS for 2h followed by stimulation with 3 or 17 mM glucose, and insulin secretion into the media was measured [16]. The amount of insulin released into the media was quantified using an insulin EIA kit (Alpco Diagnostics, Windham, NH, USA) according to the manufacturer’s instructions and was normalized to the amount of protein.

### Measurement of serum Ca^2+^ level

Mice were fasted for 18 h and blood samples were collected. After centrifugation at 3000 g for 20 min, the supernatant was used to measure serum Ca^2+^ levels using the AU Calcium oCPC reagent (Beckman Coulter, Krefeld, Germany). Briefly, Ca^2+^ ions were reacted with o-Cresolphthalein-compex one (oCPC) to form an intense purple colored Ca^2+^-oCPC complex, and the intensity was measured using a Beckman Coulter AU480 analyzer (Beckman Coulter, Krefeld, Germany).

### Intracellular calcium level measurement

Changes in intracellular calcium during glucose stimulated insulin secretion (GSIS) was determined with the Fluo-4 NW calcium Assay kit (Molecular Probes, Eugene, OR, USA). Briefly, cells were plated and 3 mM or 17 mM glucose/ in HBSS was loaded with Fluo-4 NW in the presence of probenecid (4-dipropylamino-sulfonyl benzoic acid), and fluorescence (494 nm excitation, 516 nm emission) was measured.

### Quantitative real-time PCR analysis

Total RNA was isolated from isolated islets or INS-1 cells, and cDNA was synthesized using a PrimeScript 1st strand cDNA synthesis kit (Takara). Quantitative real-time PCR was performed using the Power SYBR Green Master Mix (Applied-Biosystems) and Applied Biosystem Prism 7900HT sequence detection system. PCR was carried out and stopped at 40 cycles (2 minutes at 50°C, 10 minutes at 95°C, and 40 cycles of 10 seconds at 95°C and 1 minute at 60°C). The primer sequences used were mouse CaSR F: GCACAGTTGCCTTGTGATCC, R: GAGTCCTCCTGTCTGCGATG; rat CaSR F: TGTAACACCGTCTCCAAGGC, R: GCAGAACTCGTCCAGGTTCA; mouse Granuphilin F: GCCTGGGCCGCTTAATTC, R: TCGGCACACTAAATGATTGCA; mouse Cyclophilin F: TGGAGAGCACCAAGACAGAC, R: TGCCGGAGTCGACAATGAT; rat Cyclophilin F: GGTCTTTGGGAAGGTGAAAGAA, R: GCCATTCCTGGACCCAAAA; mouse Pdx-1 F: GAAATCCACCAAAGCTCACG, R: CGGGTTCCGCTGTGTAAG; mouse Glut-2 F: CCTCAAGAGGTAATAATATCCC, R: CCATCAAGAGGGCTCCAGTC. The relative copy number was calculated using the threshold crossing point (Ct) and the ΔΔCt method.

### Statistical Analysis

Results are expressed as mean ± S.D. of three separate experiments. ANOVA followed by Scheffe’s test for multiple comparisons was used to determine the significance of any differences between groups. *P*<0.05 was considered significant.

## Results

### Changes in body weight, food intake, and non-fasting blood glucose levels during aging in C57BL/6 mice

**Fig 1:**
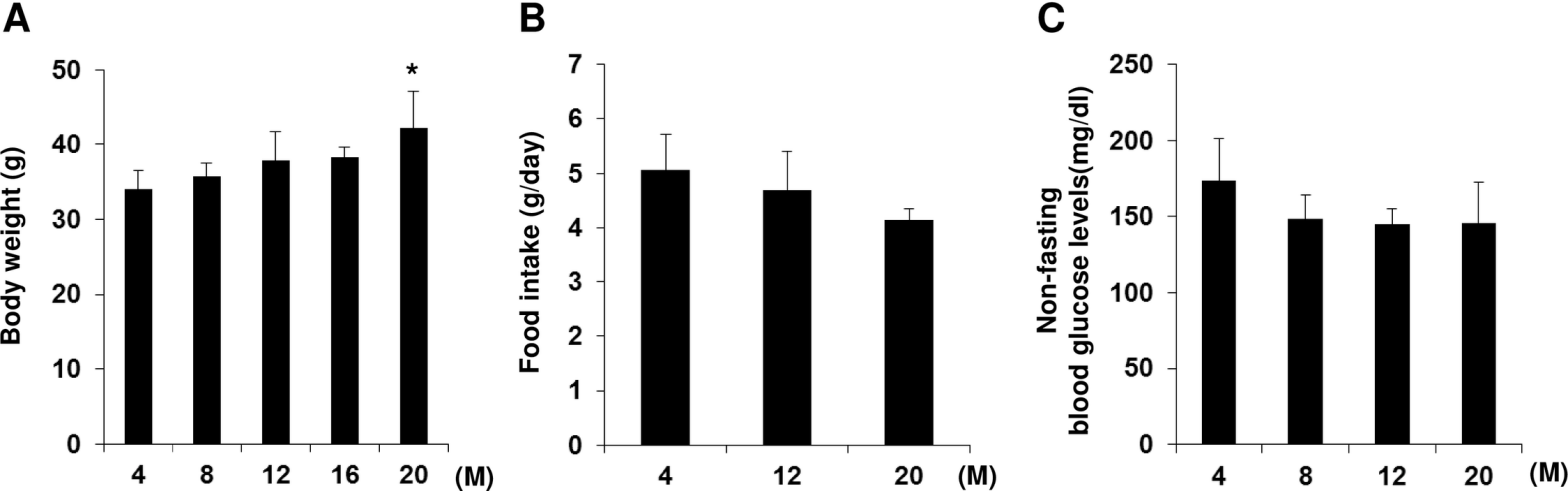
Changes in body weight, food intake, and non-fasting blood glucose levels during aging in C57BL/6 mice. Different groups of mice at different ages (4, 8, 12, 16 and 20-months old) were fed with regular chow diet, and (A) body weight, (B) food intake and (C) non-fasting blood glucose levels were measured. n = 7–12 per group. Values are means ± SD. *p<0.05 *vs*. 4-month-old mice.

Body weight and food intake was measured in different groups of C57BL/6J mice at different ages (from 4 to 20 months). A gradual increase in body weight was observed in mice during aging (Fig 1A), even though the amount of food intake was not significantly different (Fig 1B). To determine whether blood glucose level was affected by the aging process, we monitored non-fasting blood glucose levels and found that they were not significantly changed during aging (Fig 1C).

### Glucose and insulin tolerance tests in different ages of mice

**Fig 2:**
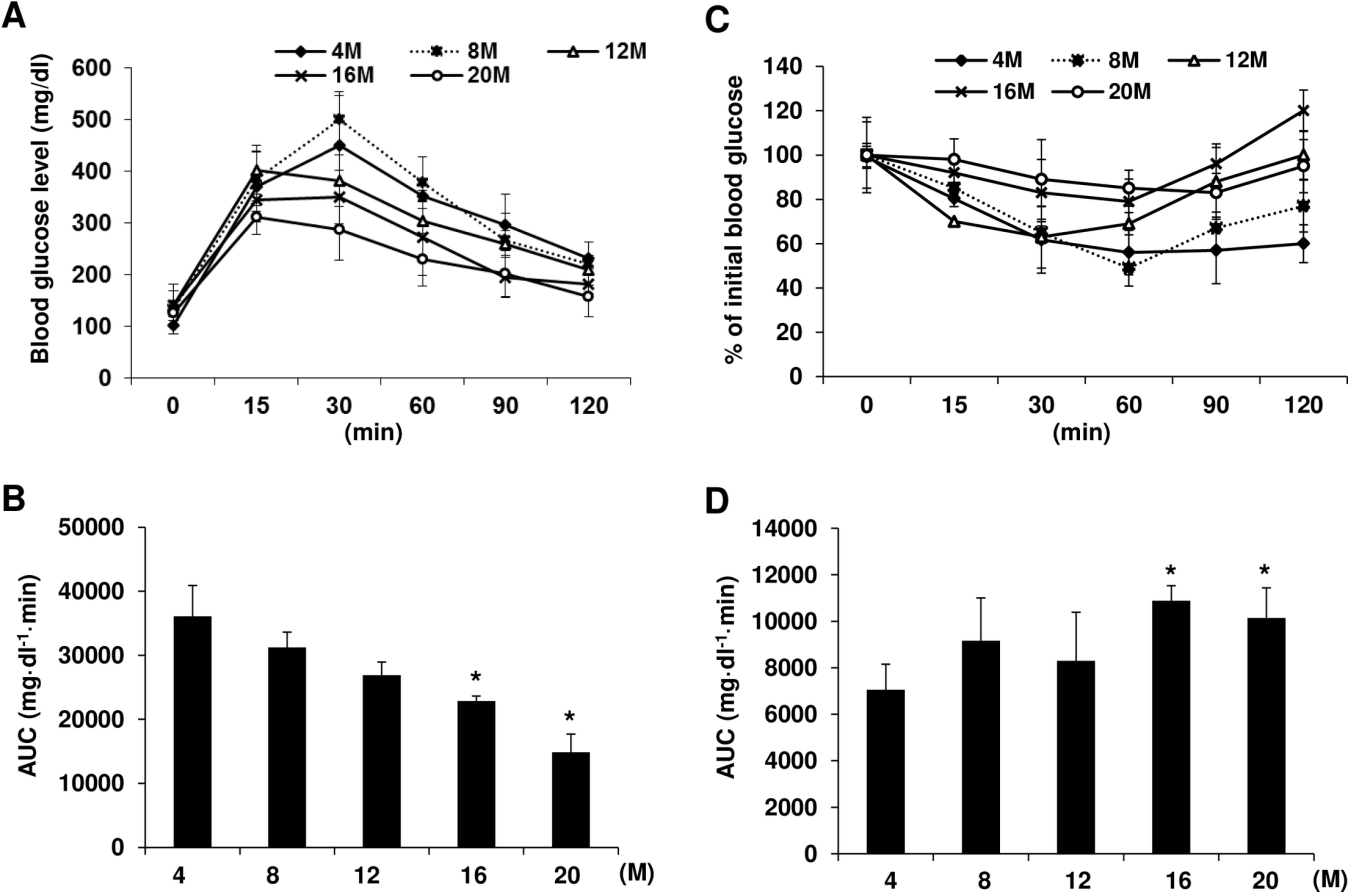
Glucose tolerance and insulin tolerance during aging in C57BL/6 mice. (A) Glucose tolerance tests (GTT) were performed in different groups of mice at different ages (4, 8, 12, 16, and 20 months of age) by intraperitoneal injection of glucose (2 g/kg, i.p.) and changes in blood glucose concentration were monitored at the indicated time points. (C) Insulin tolerance tests (ITT) were performed after insulin injection (1 U/kg, i.p.). Area under the curve (AUC) was calculated as mg·dl^-1^·min based on the GTT (B) and ITT curves (D). n = 5–7 per group, Data are means ± SD. *p < 0.05 *vs*. 4-month-old mice.

We performed glucose tolerance tests in different groups of mice at different ages (4, 8, 12, 16 and 20 months) and found that glucose tolerance appeared to increase with aging until 20 months of age, which was the oldest age monitored in this study (Fig 2A). This resulted in a significant decrease in the area under the curve (AUC) in 16- and 20-month-old mice compared with 4-month-old mice (Fig 2B). Next, we investigated insulin responsiveness by performing insulin tolerance tests. At 16 and 20 months of age, mice showed little reduction of glucose in response to insulin compared with mice at 4 months of age (Fig 2C). AUC calculation showed a significant increase in the aged group (16 and 20-month old mice) compared with the young group (4-month old mice) (Fig 2D), indicating reduced insulin sensitivity with age.

### Morphological changes in pancreatic islets during aging

**Fig 3:**
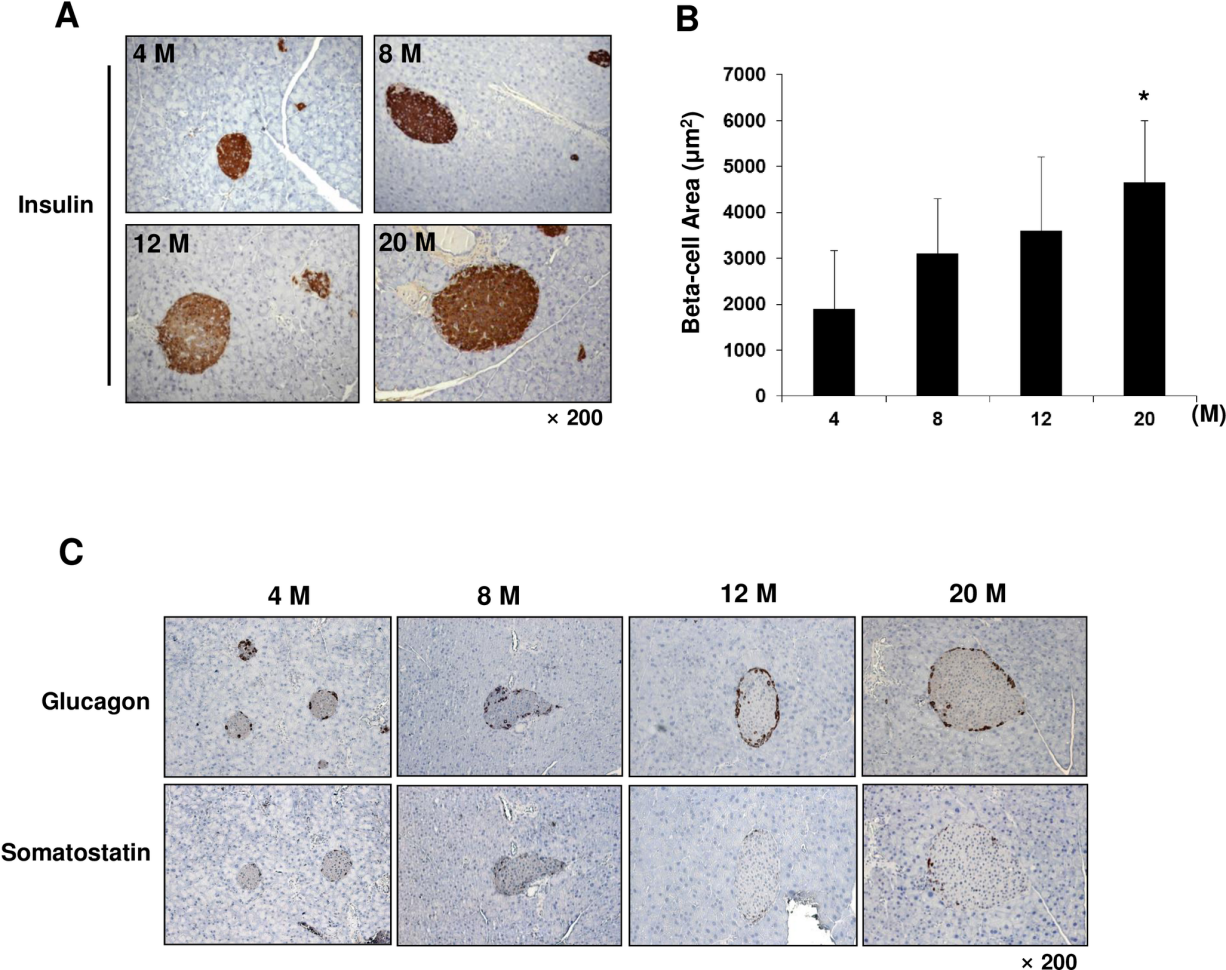
Insulin-, glucagon- and somatostatin-positive cells in pancreatic islets during aging in C57BL/6 mice. (A) Beta cells in pancreatic islets from different ages (4, 8, 12, and 20 months) of mice were examined by immunohistochemical staining with an antibody against insulin. Representative images are shown at × 200 magnification. (B) The mean beta cell area was calculated by dividing the area of all insulin-positive cells by the number of islets. (C) Alpha and delta cells in pancreatic islets from different ages of mice were examined with antibodies against glucagon and somatostatin. Representative images are shown at × 200 magnification. n = 6 per group, Data are mean ± SD. * p <0.05 *vs*. beta cell area in 4-month-old mice.

To investigate whether there are any changes in the beta cell area during aging, we examined insulin-positive cells by staining pancreatic sections with anti-insulin antibody in 4, 8, 12, and 20-month-old mice. As shown in Fig 3A, the islets had a regular shape with beta cells located in the center. Quantitative analysis of insulin-positive cells demonstrated that the beta cell area gradually increased with aging, and was significantly increased in 20-month-old mice (Fig 3B). Next, we checked the distribution of non-beta cells in the islets and found that glucagon- and somatostatin-secreting cells were located in the periphery, and this pattern was maintained during the aging process (Fig 3C).

### Insulin secretion and beta cell specific gene expression in islets from mice of different ages

**Fig 4:**
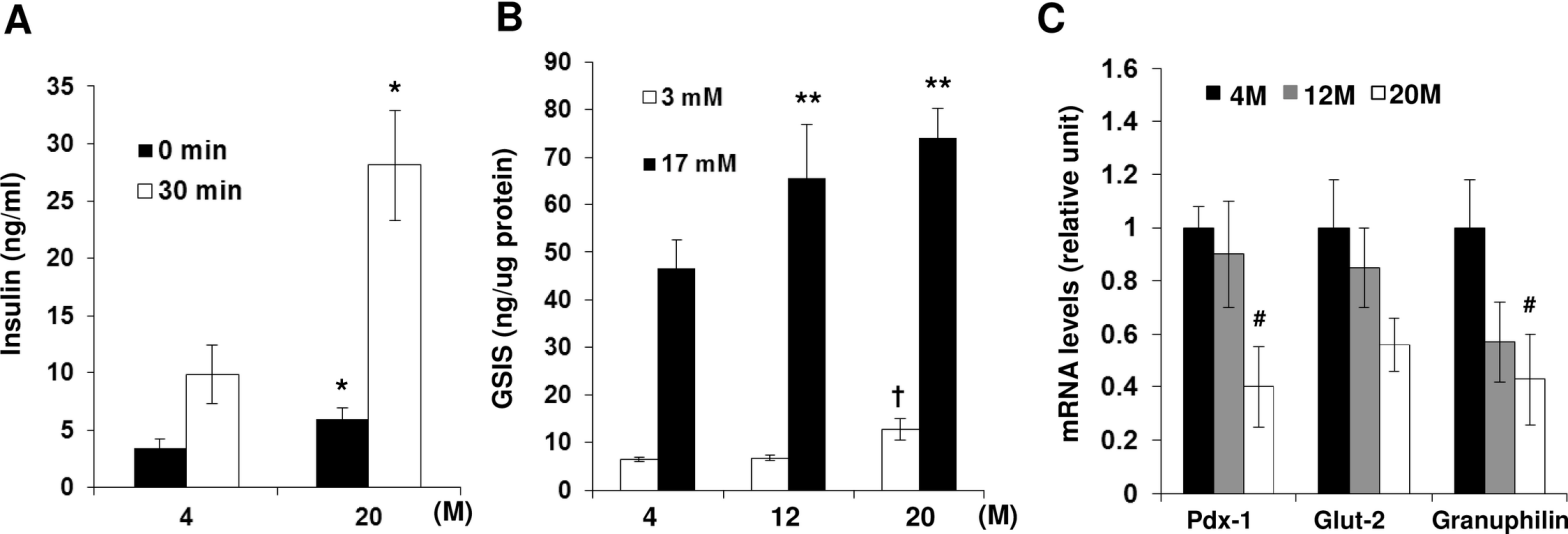
Glucose-stimulated insulin secretion and mRNA expression in islets during aging in C57BL/6 mice. (A) Serum insulin levels in 4- and 20-month-old mice. Blood samples were collected at the indicated time points and serum insulin levels were measured by insulin ELISA assay kit. (B) Islets were incubated with HEPES balanced salt solution for 2 h followed by stimulation with low (3 mM glucose) or high glucose (17 mM glucose), and insulin secretion into the media was measured. (C) Isolated islets from mice of each age were harvested and mRNA expression of Pdx-1, Glut-2, and granuphilin was analyzed by quantitative real-time PCR using specific primers. Relative expression was normalized to cyclophilin gene. Data are expressed as mean ± SD. n = 3–4 per group, *p<0.05 *vs* 4-month-old mice at the same time point, **p<0.05 *vs*. 17 mM glucose-treated islets from 4-month-old mice, †p<0.05 *vs*. 3 mM glucose-treated islets from 4-month-old mice, # p<0.05 *vs*. islets from 4-month-old mice.

To investigate whether there are any changes in insulin secretion during aging, we measured basal and glucose-stimulated serum insulin levels in 4 and 20-month-old mice. As shown in Fig 4A, 20-month-old mice showed an significant increase in circulating insulin levels compared with 4-month-old mice both when fasting and 30 min after glucose loading (Fig 4A). Next, we examined insulin secretion in response to glucose using isolated islets from 4, 12, and 20-month-old mice. Insulin release at low glucose (3 mM) was similar between 4- and 12-month-old mice, but was significantly elevated in islets from 20-month-old mice. Insulin secretion at high glucose (17 mM) was significantly enhanced in 12- and 20-month-old mice compared with 4-month-old mice (Fig 4B). We next examined mRNA expression levels of genes which are involved in insulin expression and insulin secretion, such as pancreatic and duodenal homeobox 1 (Pdx-1), glucose transporter 2 (Glut-2) and granuphilin in islets from these mice. The expression of Pdx-1 mRNA transcript in the aged group (20 months) was significantly lower than in the young group (4 months). Glut-2 mRNA level appeared to decrease with aging, but was not significant. The expression level of granuphilin, a negative regulator of insulin exocytosis [17], was significantly decreased in islets from 20-month-old mice compared with that of islets from 4-month-old mice (Fig 4C).

### Serum Ca levels and CaSR expression in islets during the ageing process

**Fig 5:**
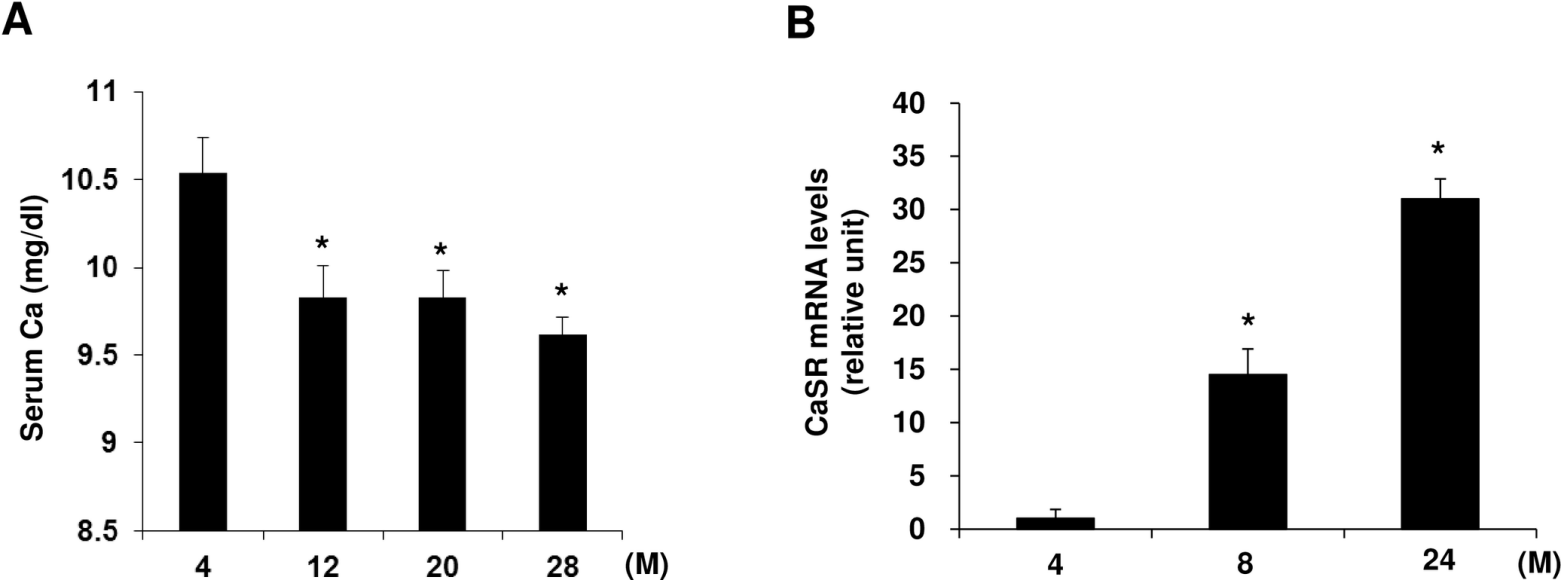
Serum calcium levels and calcium sensing receptor mRNA expression in islets during aging in C57BL/6 mice. (A) Serum was collected from 4, 12, 20 and 28-month-old mice and calcium levels were analyzed. (B) Islets were isolated from 4, 8, and 24-month-old mice and mRNA expression of calcium sensing receptor (CaSR) was examined by qRT-PCR. Relative expression was normalized to the cyclophilin gene. n = 3 per group, Data represent the mean ± SD. *p<0.05 *vs*. 4-month-old mice.

Since altered calcium homeostasis is correlated with glucose tolerance and beta cell function [18], we investigated whether serum Ca^2+^ levels are changed during aging. As shown in Fig 5A, serum calcium levels were significantly reduced in 12-, 20- and 28-month-old mice compared with 4-month old mice. Next, we examined the mRNA expression of CaSR, which is known to be involved in insulin secretion [14] and found that CaSR mRNA expression was significantly increased at 8 months and up to 24 months of age (Fig 5B). When we checked the localization of CaSR in insulin-positive cells (S1 Methods), we found that CaSR was abundantly expressed in pancreatic islets as previously reported [14], and we confirmed that most CaSR immunoreactivity was colocalized with insulin-positive cells in islets of mice at all ages (S1 Fig).

### Effect of Ca^2+^ depletion on CaSR expression and glucose stimulated insulin secretion in INS-1 cells and mouse islets

**Fig 6:**
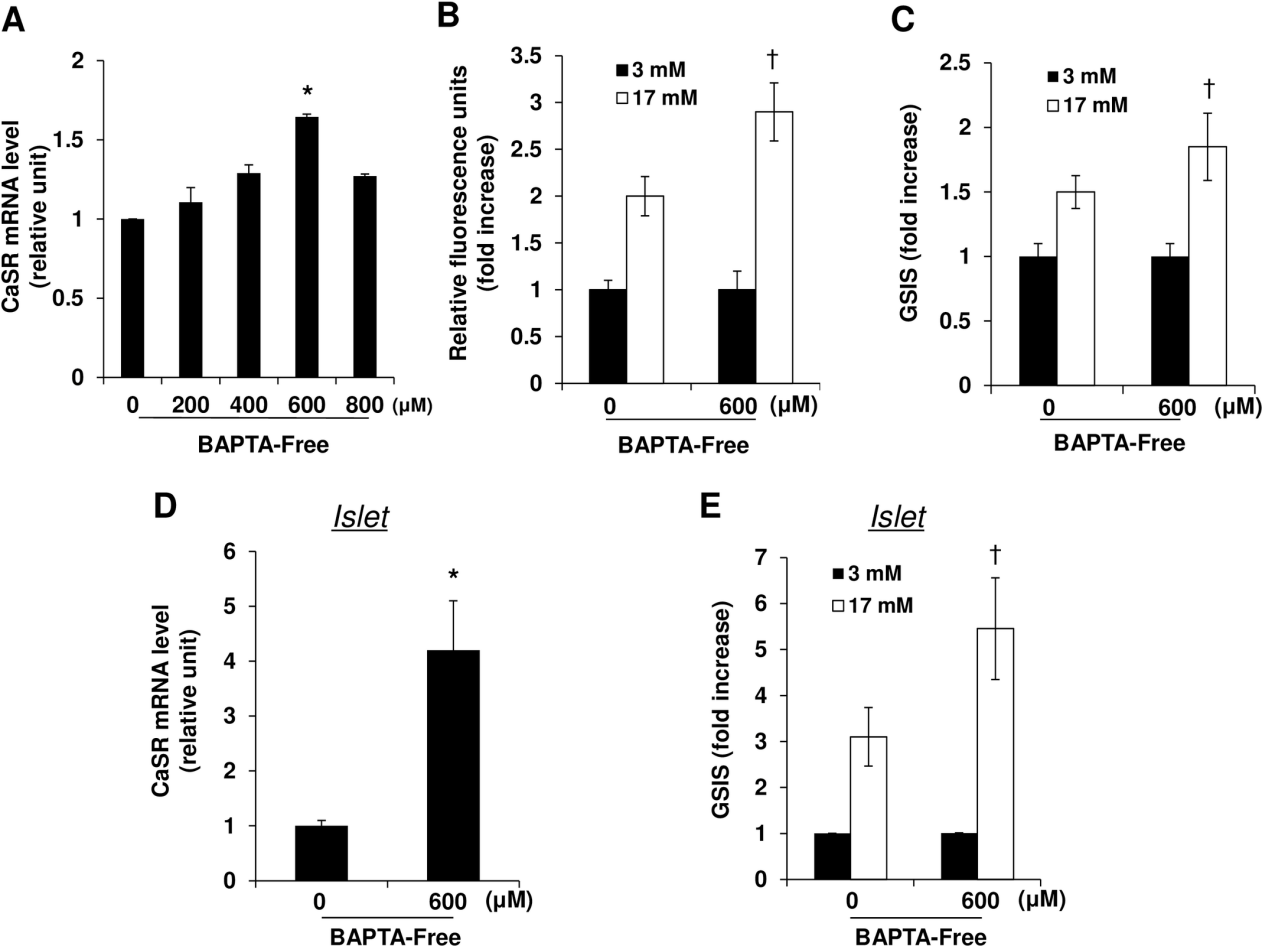
Effect of Ca^2+^ depletion on calcium sensing receptor (CaSR) mRNA expression and GSIS in INS-1 cells and mouse islets. (A) INS-1 cells were treated with the indicated concentrations of the free acid form of BAPTA (BAPTA-Free). After 24 h, cells were harvested and mRNA expression of CaSR was examined by qRT-PCR. Relative expression was normalized to the cyclophilin gene. (B,C) INS-1 cells were treated with 600 μM BAPTA-Free for 24 h and then incubated in 3 mM or 17 mM glucose. (B) Changes in intracellular calcium were measured by fluorescence. Data are represented as the increase in fluorescence versus the basal value (3 mM glucose). (C) The amount of insulin released into the supernatant was quantified using an insulin EIA kit and normalized to the amount of total protein. (D,E) Isolated mouse islets were treated with BAPTA-Free (600 μM) for 24 h. (D) mRNA levels of CaSR were analyzed by qRT-PCR and (E) the insulin secretion responses to glucose were analyzed. (C, E) The data are expressed as the increase in GSIS versus the basal value (3 mM glucose). Data represent the mean ± SD for three independent experiments. *p<0.05 *vs*. non-treated cells, †p<0.05 *vs*. 17 mM glucose treated cells without BAPTA-Free.

As we found that lower serum Ca^2+^ levels were correlated with higher CaSR mRNA expression and higher insulin secretion in islets, we tried to confirm this correlation in INS-1 cells and isolated mouse islets. To decrease extracellular Ca^2+^ levels, INS-1 cells were treated with various concentrations (0 ~ 800 μM) of the free acid form of BAPTA (1,2-bis(o-aminophenoxy)ethane-N,N,N’,N-tetraacetic acid) (BAPTA-Free), an extracellular Ca^2+^ chelating agent [19], for 24 h and CaSR levels and insulin secretion was measured. As shown in Fig 6A, a significant increase of CaSR mRNA was observed at 600 μM BTAPTA-Free treatment. As well, 600 μM BAPTA-Free treatment increased intracellular Ca^2+^ levels in response to high glucose concentration (Fig 6B) and resulted in enhanced GSIS (Fig 6C) compared with untreated INS-1 cells. We also confirmed that BAPTA-Free treatment also increased CaSR mRNA expression (Fig 6D) and insulin secretion (Fig 6E) in mouse islets. To investigate whether intracellular Ca^2+^ also affects the CaSR expression and GSIS, INS-1 cells were treated with 0 ~ 600 μM concentrations of BAPTA-AM (acetoxymethyl), an intracellular Ca^2+^ chelating agent [20] and CaSR mRNA expression was examined. As shown in S2 Fig, CaSR mRNA expression was increased by BAPTA-AM in a dose-dependent manner, peaked at 100 μM, and gradually decreased with higher doses of BAPTA-AM. Insulin secretion in response to high glucose was significantly increased when INS-1 cells were treated with 50 or 100 μM of BAPTA-AM, but treatment with 200 μM or 400 μM BAPTA did not further increase insulin secretion. A higher concentration of BAPTA-AM (600 μM) downregulated CaSR expression and consequently reduced insulin secretion (S2 Fig). To confirm the correlation of CaSR expression and insulin secretion, we transfected INS-1 cells with a CaSR plasmid (S1 Methods). We found that CaSR mRNA was increased and insulin secretion in response to high glucose was enhanced compared with vector-transfected cells (S3 Fig).

## Discussion

Normal aging is associated with a progressive deterioration in most endocrine functions, which may be responsible for metabolic disorders [21]. Defective glucose homeostasis during the aging process is well recognized, but the underlying biological mechanisms are still not clearly elucidated. Therefore, an understanding of the changes that occur with age might be helpful in elucidating the mechanism of aging-related metabolic syndromes.

Maintenance of normal glucose homeostasis during the aging process is correlated with decreased weight gain [22]. In this study, we found that body weight gain increased with age, despite decreased food intake. Silver et al. reported that with the aging process, metabolic imbalances such as an increase in body fat occur, despite a decline in food intake [23], and these are caused by a decline in physical activity and resting metabolic rate [24].

Many studies have reported increasing insulin resistance and declining glucose tolerance concomitant with abnormal insulin secretion as characteristics of aging in both humans and experimental animals. Old Wistar rats (24 months) and Sprague-Dawley rats (28 months) display oral glucose intolerance and exhibit a decrease in a glucose-stimulated insulin release compared with young rats (2–4 months) [9, 25]. Elderly healthy men (67–80 years) also show deterioration in glucose homeostasis as evidenced by insulin resistance, defective insulin secretion, and insulin action [26–29]. However, some reports observe no change in insulin sensitivity and beta cell response in elderly subjects or impaired glucose metabolism in young subjects, suggesting that the decline of glucose homeostasis is not caused by the aging process. For example, normal mice of the Aston strain show impaired disposal after glucose load in young mice at 10 weeks of age [30] and inbred C57BL/KsJ mice also show severe glucose intolerance in the early pre-weaning period [31]. Moreover, age-dependent improvement in glucose tolerance and increase in insulin levels are observed in C57BL/6J and KK-Ay mice [32, 33]. In our study, we found that glucose tolerance improved with age at least up to 20 months. Some human studies have demonstrated that there was no decrease in insulin sensitivity and beta cell function in healthy elderly subjects [34, 35], and that older normoglycemic individuals had similar insulin responses compared with younger adults [36]. These reports including our results suggest that defects of glucose homeostasis are not always the result of the aging process.

Under insulin-resistant conditions, an increase in beta cell mass can compensate for the increased demand to maintain normal glucose homeostasis [37]. In our study, results obtained both in vitro and in vivo demonstrated that beta cell function and beta cell number markedly increased in response to age-dependent insulin resistance, suggesting that beta cell compensation during the aging process promotes the maintenance of normal glucose levels in aged mice. However, at the oldest age studied (over 30 months), the beta cell areas were decreased, insulin levels were reduced, and glucose intolerance was observed, indicating a failure of beta cell compensation (unpublished data). Nevertheless, in ages up to 20 months, the increase in insulin secretion is able to compensate for the insulin resistance, and these results in the increase in glucose tolerance seen in C57BL/6J mice.

The adaptive capacity of the beta cell mass and function depends on the activity of transcriptional and translational regulators, which tightly modulate the expression of related genes. Pdx-1 and Glut-2 are major regulators for beta-cell function [38, 39], and their expression levels were decreased with age in our study. As a reduction of Pdx-1 and Glut-2 in beta cells impairs GSIS, we speculated that the genes involve in insulin exocytosis might play a role in increased beta cell function with aging seen in our study. We found that mRNA expression of granuphilin/Slp, which is a negative regulator of insulin exocytosis [17], was decreased during aging. Overexpression of granuphilin in beta cell line inhibited insulin secretion, whereas granuphilin-null islets exhibited increased insulin secretion [40, 41]. Kato et al. reported that the mRNA level of granuphilin was increased in islets from several diabetic mouse models and in palmitate-treated normal islets, accompanied by reduction in insulin secretion [42]. Therefore, downregulation of granuphilin in aged islets might play an important role to compensate for the reduced expression of Pdx-1 and Glut-2 and subsequently increasing GSIS.

There are contradictory reports about serum calcium levels and the risk for type 2 diabetes. Some studies report that serum total calcium levels are higher in diabetic individuals than in normal people [43–45] and are positively associated with impairment of glucose tolerance and insulin resistance [18, 46]. In contrast, other studies demonstrate that elevated serum calcium levels are not associated with insulin secretion and insulin resistance [47, 48]. The role of serum calcium in diabetes development is unclear, but our data suggests that reduced Ca^2+^ levels in aged mice are associated with increased insulin secretion and glucose tolerance.

Extracellular CaSR is one of the factors that regulate systemic Ca^2+^ homeostasis, and is expressed in tissues related to calcium control such as kidney, intestine, bones and islets [49]. CaSR plays a role in cell-to-cell communication within the pancreatic islets by detecting local concentrations of extracellular Ca^2+^ (co-released with insulin) in the intra-islet space and stimulating the signal across the islets resulting in enhanced glucose induced insulin secretion [14]. We found that expression of CaSR mRNA was increased with age, which would be expected to promote insulin secretion. We also demonstrated that Ca^2+^ depletion both in intracellular and extracellular stores can induce CaSR mRNA expression, and increased CaSR expression was positively correlated with enhanced GSIS in INS-1 cells and mouse islets. As it was reported that treatment with a CaSR agonist increases intracellular Ca^2+^ levels in MIN-6 cells [14], upregulation of CaSR might increase intracellular Ca^2+^ levels and result in increased insulin secretion in aged mice. However, when both intracellular and extracellular Ca^2+^ was completely depleted with high concentrations of BAPTA-Free or BAPTA-AM, beta cell viability might be decreased and glucose-stimulated insulin secretion is not increased further, although the expression level of CaSR is increased, and eventually GSIS is decreased. There results suggest that prolonged Ca^2+^ depletion in the serum of very old mice might eventually result in defective insulin secretion. Although the exact mechanism of insulin secretion induced by CaSR upregulation is not known in the present study, it is possible that CaSR-linked phospho lipase C activation generates inositol triphosphate to liberate Ca^2+^ stored in the endoplasmic reticulum, and p42/44 MAPK activation might be involved in insulin secretion [13].

Our results indicate that increased insulin resistance is associated with aging, and beta cell compensation occurs to maintain glucose homeostasis. Enhanced beta cell function regulated by CaSR expression as well as increased number of beta cells might be mechanisms for age-related beta cell compensation, providing new insights into maintenance of glucose homeostasis during aging.

## Supporting Information

S1 Fig Colocalization of calcium sensing receptor (CaSR) and insulin-positive cells in islets from mice of different ages. Pancreatic sections were prepared from mice of different ages (4, 8, 12, and 20 months) and stained with DAPI (blue), insulin (red), CaSR (green) antibodies. Scale bar = 20 μm.
(TIF)

S2 Fig Effect of intracellular Ca^2+^ depletion on calcium sensing receptor (CaSR) mRNA expression and glucose-stimulated insulin secretion in INS-1 cells. (A) INS-1 cells were treated with the indicated concentrations of BAPTA-AM. After 24 h, cells were harvested and mRNA expression of CaSR was examined by qRT-PCR. Relative expression was normalized to the cyclophilin gene. (B) Cells were treated with various concentrations of BAPTA-AM for 24 h and then incubated in 3 mM or 17 mM glucose with or without BAPTA-AM for 2 h. The amount of insulin released into the supernatant was quantified using an insulin EIA kit and normalized to the total protein amount. The data are expressed as the increase in GSIS versus the basal value (3 mM glucose). Data represent the mean ± SD for three independent experiments. *p<0.05 *vs*. non-treated cells, **p<0.005 *vs*. non-treated cells, †p<0.05 *vs*. 17 mM glucose-treated cells without BAPTA-AM.
(TIF)

S3 Fig Effect of insulin secretion in CaSR overexpressing INS-1 cells. (A) Myc-CaSR (CaSR) or control vector (CON) was transfected into INS-1 cells, and CaSR mRNA levels were analyzed by qRT-PCR. (B) At 24 h after CaSR transfection, glucose-stimulated insulin secretion was examined. The data are expressed as the increase in GSIS versus the basal value (3 mM glucose). Data represent the mean ± SD for three independent experiments. *p < 0.05 *vs*. 17 mM glucose-treated cells with CON vector.
(TIF)

S1 Methods Immunofluorescence staining and CaSR overexpression. For immunofluorescence staining, pancreatic sections were deparaffinized in toluene, dehydrated in alcohol, and washed in water. After the antigen retrieval process, non-specific protein binding sites were saturated with blocking solution. Tissue sections were incubated with primary antibodies (rabbit anti-insulin, 1:100; mouse anti-CaSR, 1:100) overnight in a cold room and then washed with PBS. Sections were incubated with fluorescein isothiocyanate-conjugated anti-mouse and rhodamine-conjugated anti-rabbit secondary antibodies for 30 min. Nuclei were then fluorescently labeled with DAPI. The labeled cells were observed under a confocal microscope. For CaSR overexpression, pCMV6-CaSR plasmid DNA was purchased from Origene (Rockville, MD, USA). INS-1 cells were transfected with pCMV6-empty vector or pCMV6-CaSR plasmid DNA using Lipofectamine 2000 reagent (Invitrogen, Garlsbad, CA, USA) according to the manufacturer’s instruction.
(DOCX)
