# Supplementary figures and images for: Increase of Calcium Sensing Receptor Expression Is Related to Compensatory Insulin Secretion during Aging in Mice
Source: PLoS One. 2016 Jul 21;11(7):e0159689. doi: 10.1371/journal.pone.0159689 (PMC4956240; doi:10.1371/journal.pone.0159689)

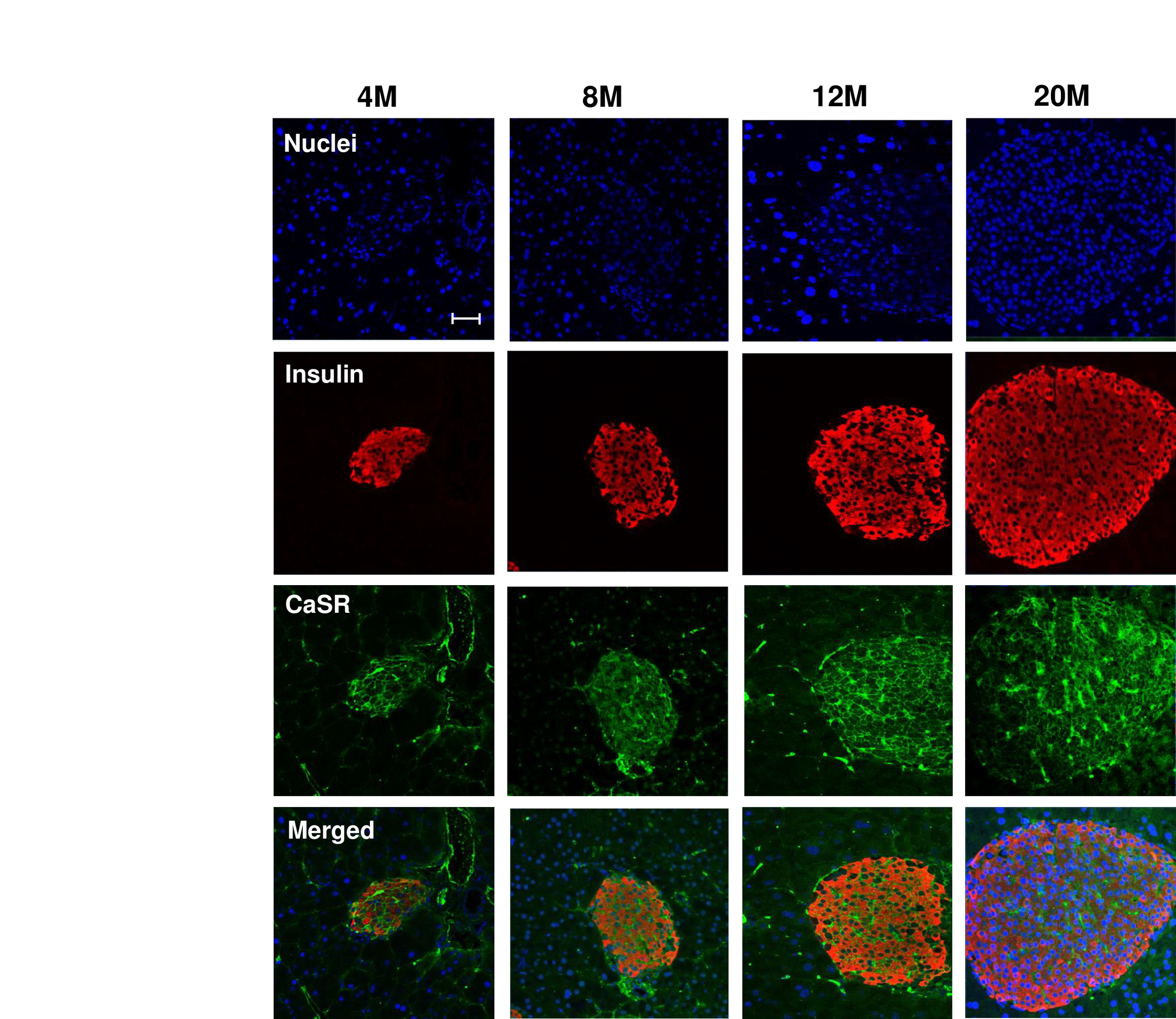

Supplement: S1 Fig — Pancreatic sections were prepared from mice of different ages (4, 8, 12, and 20 months) and stained with DAPI (blue), insulin (red), CaSR (green) antibodies. Scale bar = 20 μm. (TIF) [file pone.0159689.s001.tif]

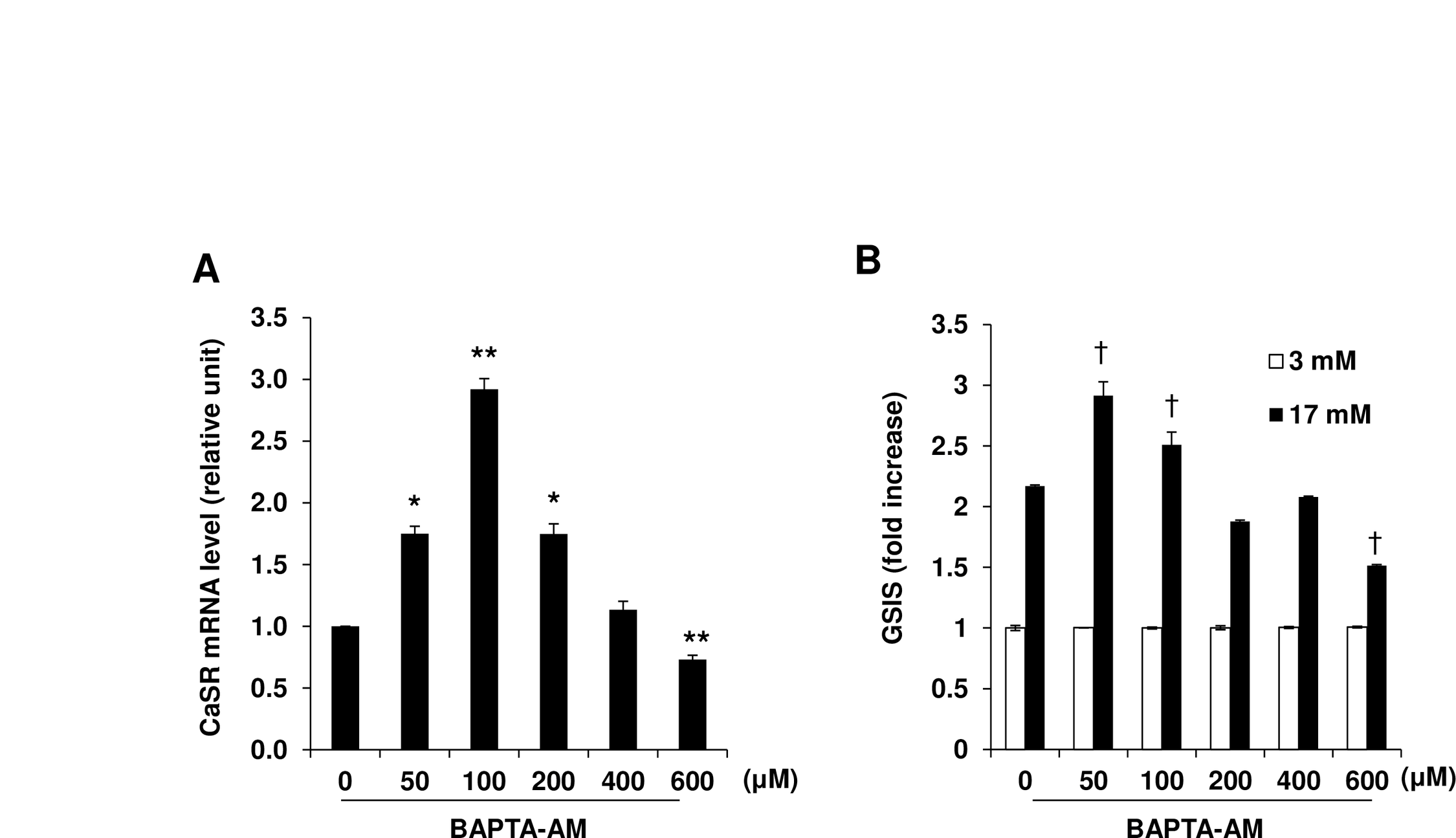

Supplement: S2 Fig — (A) INS-1 cells were treated with the indicated concentrations of BAPTA-AM. After 24 h, cells were harvested and mRNA expression of CaSR was examined by qRT-PCR. Relative expression was normalized to the cyclophilin gene. (B) Cells were treated with various concentrations of BAPTA-AM for 24 h and then incubated in 3 mM or 17 mM glucose with or without BAPTA-AM for 2 h. The amount of insulin released into the supernatant was quantified using an insulin EIA kit and normalized to the total protein amount. The data are expressed as the increase in GSIS versus the basal value (3 mM glucose). Data represent the mean ± SD for three independent experiments. *p<0.05 vs. non-treated cells, **p<0.005 vs. non-treated cells, †p<0.05 vs. 17 mM glucose-treated cells without BAPTA-AM. (TIF) [file pone.0159689.s002.tif]

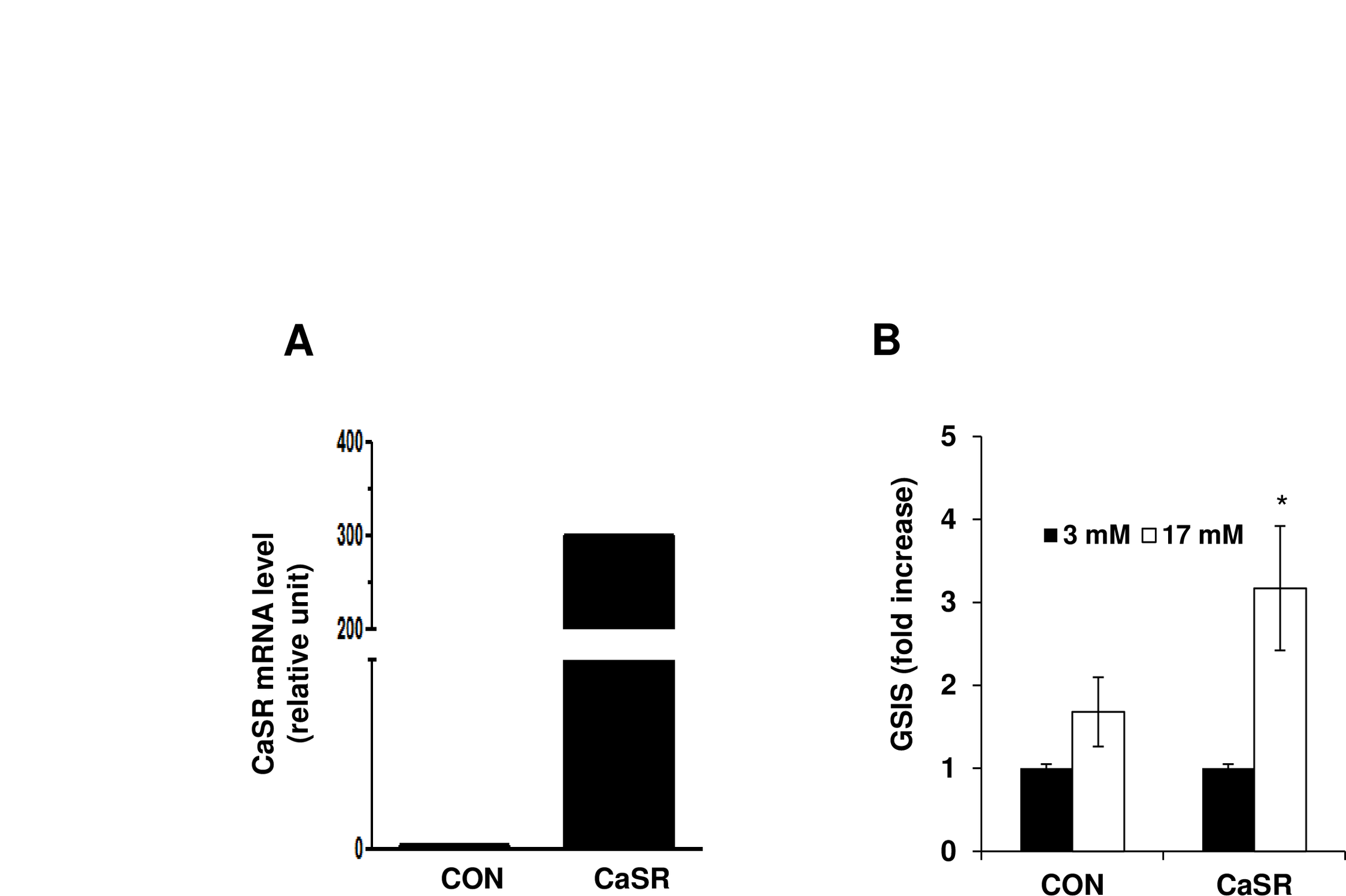

Supplement: S3 Fig — (A) Myc-CaSR (CaSR) or control vector (CON) was transfected into INS-1 cells, and CaSR mRNA levels were analyzed by qRT-PCR. (B) At 24 h after CaSR transfection, glucose-stimulated insulin secretion was examined. The data are expressed as the increase in GSIS versus the basal value (3 mM glucose). Data represent the mean ± SD for three independent experiments. *p < 0.05 vs. 17 mM glucose-treated cells with CON vector. (TIF) [file pone.0159689.s003.tif]
