# Supplementary material for: Increase of Calcium Sensing Receptor Expression Is Related to Compensatory Insulin Secretion during Aging in Mice
Source: PLoS One. 2016 Jul 21;11(7):e0159689. doi: 10.1371/journal.pone.0159689 (PMC4956240; doi:10.1371/journal.pone.0159689)
Supplement: S1 Methods — For immunofluorescence staining, pancreatic sections were deparaffinized in toluene, dehydrated in alcohol, and washed in water. After the antigen retrieval process, non-specific protein binding sites were saturated with blocking solution. Tissue sections were incubated with primary antibodies (rabbit anti-insulin, 1:100; mouse anti-CaSR, 1:100) overnight in a cold room and then washed with PBS. Sections were incubated with fluorescein isothiocyanate-conjugated anti-mouse and rhodamine-conjugated anti-rabbit secondary antibodies for 30 min. Nuclei were then fluorescently labeled with DAPI. The labeled cells were observed under a confocal microscope. For CaSR overexpression, pCMV6-CaSR plasmid DNA was purchased from Origene (Rockville, MD, USA). INS-1 cells were transfected with pCMV6-empty vector or pCMV6-CaSR plasmid DNA using Lipofectamine 2000 reagent (Invitrogen, Garlsbad, CA, USA) according to the manufacturer’s instruction. (DOCX) [file pone.0159689.s004.docx]

**S1 Methods. Immunofluorescence staining and CaSR overexpression.** For immunofluorescence staining, pancreatic sections were deparaffinized in toluene, dehydrated in alcohol, and washed in water. After the antigen retrieval process, non-specific protein binding sites were saturated with blocking solution. Tissue sections were incubated with primary antibodies (rabbit anti-insulin, 1:100; mouse anti-CaSR, 1:100) overnight in a cold room and then washed with PBS. Sections were incubated with fluorescein isothiocyanate-conjugated anti-mouse and rhodamine-conjugated anti-rabbit secondary antibodies for 30 min. Nuclei were then fluorescently labeled with DAPI. The labeled cells were observed under a confocal microscope. For CaSR overexpression, pCMV6-CaSR plasmid DNA was purchased from Origene (Rockville, MD, USA). INS-1 cells were transfected with pCMV6-empty vector or pCMV6-CaSR plasmid DNA using Lipofectamine 2000 reagent (Invitrogen, Garlsbad, CA, USA) according to the manufacturer’s instruction.
